# The role of genetic and epigenetic modifications as potential biomarkers in the diagnosis and prognosis of thyroid cancer

**DOI:** 10.3389/fonc.2024.1474267

**Published:** 2024-11-04

**Authors:** Essa M. Sabi

**Affiliations:** Clinical Biochemistry Unit, Department of Pathology, College of Medicine, King Saud University, Riyadh, Saudi Arabia

**Keywords:** thyroid cancer, genetic modifications, epigenetic modifications, biomarkers, diagnosis, prognosis

## Abstract

Thyroid cancer (TC) is the most common endocrine cancer, which contributes to more than 43,600 deaths and 586,000 cases worldwide every year. Among the TC types, PTC and FTC comprise 90% of all TCs. Genetic modifications in genes are responsible for encoding proteins of mitogen-associated protein kinase cascade, which is closely related with numerous cellular mechanisms, including controlling programmed cell death, differentiation, proliferation, gene expression, as well as in genes encoding the PI3K (phosphatidylinositol 3-kinase)/protein kinase B (AKT) cascade, which has contribution in controlling cell motility, adhesion, survival, and glucose metabolism, have been associated with the TC pathogenesis. Various genetic modifications including BRAF mutations, RAS mutations, RET mutations, paired-box gene 8/peroxisome proliferator-activated receptor-gamma fusion oncogene, RET/PTC rearrangements, telomerase reverse transcriptase mutations, neurotrophic tyrosine receptor kinase fusion genes, TP53 mutations, and eukaryotic translation initiation factor 1A X-linked mutations can effectively serve as potential biomarkers in both diagnosis and prognosis of TC. On the other hand, epigenetic modifications can lead to aberrant functions or suppression of a range of signalling cascades, which can ultimately result in cancer. Various studies have observed the link between epigenetic modification and multiple cancers including TC. It has been reported that several epigenetic alterations including histone modifications, aberrant DNA methylation, and epigenetic modulations of non-coding RNAs can play significant roles as potential biomarkers in the diagnosis and prognosis of TC. Therefore, a good understanding regarding the genetic and epigenetic modifications is not only essential for the diagnosis and prognosis of TC, but also for the development of novel therapeutics. In this review, most of the major TC-related genetic and epigenetic modifications and their potential as biomarkers for TC diagnosis and prognosis have been extensively discussed.

## 1 Introduction

**Figure 1:**
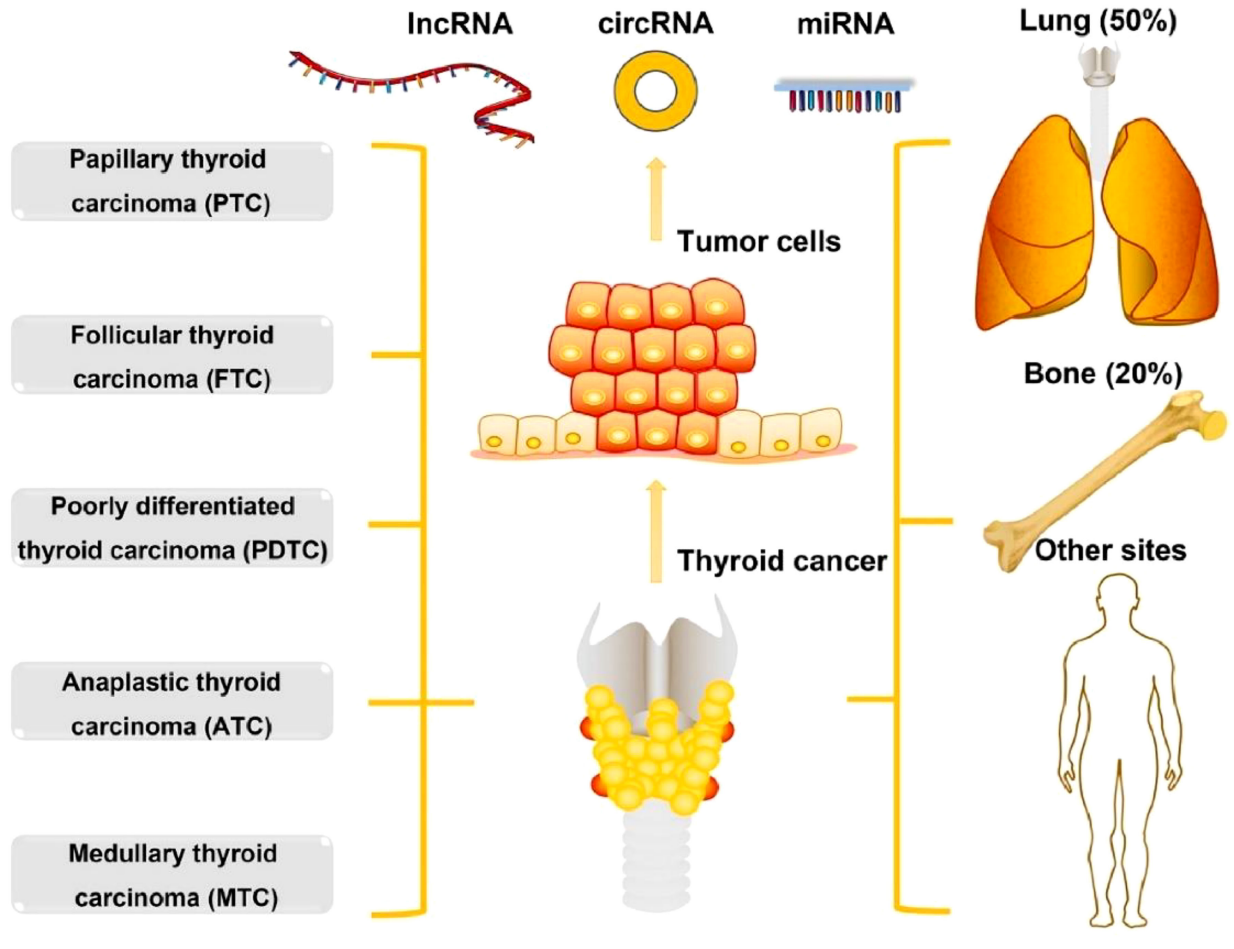
The types and metastasis of thyroid cancer. Reproduced with permission from Elsevier, (4).

Thyroid cancer (TC) is the most common endocrine cancer, which contributes to more than 43,600 deaths and 586,000 cases worldwide every year (1). In the past 30 years, the occurrence of TC has elevated in multiple developed countries (2, 3). The occurrence of TC varies by up to 15-20-fold according to geographical regions, where it is more commonly diagnosed in developed countries. High-risk regions for TC include Southern Europe, North America, New Zealand, Australia, Eastern Asia, and Polynesia. As per the histological type, TC can be classified into 5 types including anaplastic TC (ATC), medullary TC (MTC), poorly differentiated TC (PDTC), follicular TC (FTC), and papillary TC (PTC). Around 10% of TC patients contain tumour metastasis including lung and bone (20%), lung (50%), bone (25%), and other sites (5%) (**Figure 1**). PTC and FTC comprise 90% of all TCs, which generally affect people aged between 50 and 60 years (1). A range of risk factors have already been identified that can contribute to TC development including genetic predisposition, increased concentrations of thyroid-stimulating hormone, iodine excess or deficiency, and ionising radiation (5). Common diagnostic techniques of TC include histopathological evaluation of the thyroid gland tissue, fine needle aspiration cytology (FNAC), ultrasonography, and various laboratory examinations with the likelihood of estimating the tumour markers calcitonin and thyroglobulin (6). There is a growing interest in molecular genetic analysis of FNAC samples (6). In addition, there are several mutations that are explicit for specific types of carcinomas and can thus play a role in molecular testing in the preoperative period along with alteration of the diagnosis in the case of cytologically ambiguous results (6). Decreased effectiveness of radioiodine therapy or a propensity to dedifferentiate, involvement of metastatic lymph node, and signs of elevated level of tumour aggressiveness have already been linked along with some mutations (7).

MTC arises from neural crest and most of the MTC cases are sporadic (75%) while the remaining are hereditary (25%) (8). Indeed, this wide range of progression is meticulously associated with the pattern of accumulated genetic and epigenetic changes, which are associated with tumour invasion, metastasis, and differentiation. Most of the genetic changes in TC start their activities via causing the activation of metabolic pathways. It has been observed that constitutive activation of the extracellular signal-regulated kinase (ERK)/mitogen-associated protein kinase (MAPK) cascades can result in tumorigenesis and can further mediate cell division. ERK/MAPK cascade activation is an important and common process involved with the human cancer progression and initiation. Genetic abnormalities in the *RAS* gene, *BRAF* (B-Raf Proto-Oncogene, Serine/Threonine Kinase) gene, and rearranged in translation (RET)/PTC are also linked with TC. Interestingly, the occurrence of RAS gene-associated activating mutations is reliant on the tumour histology. It was observed that RAS mutations are more commonly seen in FTC as compared to PTC. A cell membrane receptor tyrosine kinase was found to be encoded by the RET proto-oncogene. This kinase’s ligands belong to the glial-cell-line derived neurotropic factor family that results in receptor dimerisation following binding, which further results in tyrosine residue autophosphorylation and starts the ERK/MAPK cascade. Functional deficiency of RET can lead to Hirschsprung’s disease, while increased activities of RET have been linked with a range of cancer types, such as MTC. Simultaneous mutations of BRAF as well as RET/PTC have been linked with PTC (9). Interestingly, BRAF V600E mutation is limited to anaplastic, papillary, and poorly differentiated TC (10). In this review, the genetic and epigenetic basis of TC as well as the main TC-associated genetic and epigenetic modifications and their potential as biomarkers for both diagnosis and prognosis of TC have been extensively covered.

## 2 Genetic and epigenetic basis of thyroid cancer

**Figure 2:**
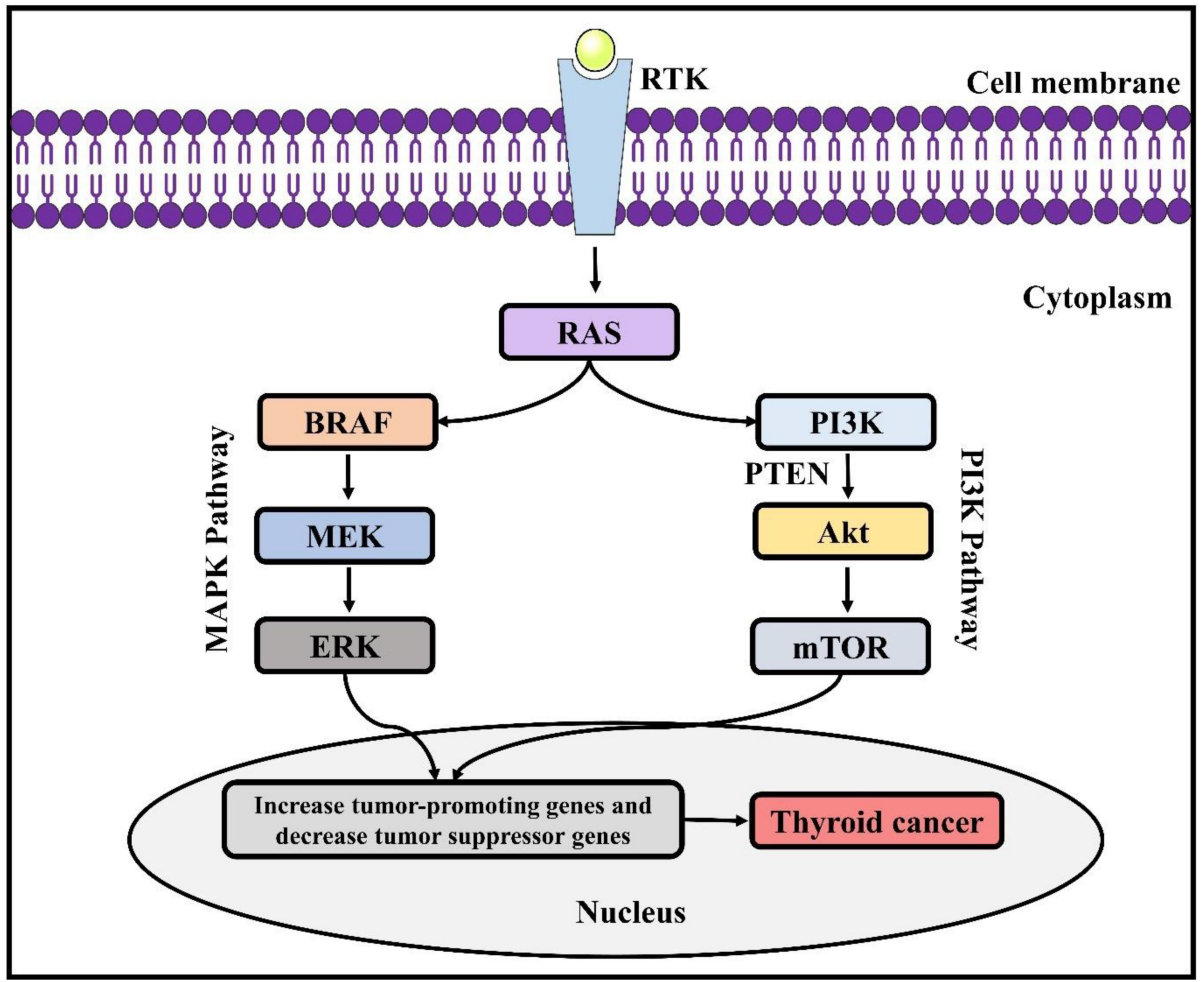
Genetic alterations involved in thyroid cancer. RAS, Rat sarcoma virus; ERK, extracellular signal-regulated kinase; BRAF, B-Raf proto-oncogene; PI3K, phosphatidylinositol 3-kinase; MAPK, mitogen-associated protein kinase; PTEN, phosphatase and tensin homolog; AKT, protein kinase B; mTOR, mammalian target of rapamycin.

Tumour progression and transformation involve the disturbance of cell signal mechanisms that control the balance between apoptosis and cell proliferation (11). Genetic modifications in genes are responsible for encoding proteins of MAPK cascade (**Figure 2**), which is closely linked with numerous cellular mechanisms, including controlling programmed cell death, differentiation, proliferation, gene expression, as well as in genes encoding the PI3K (phosphatidylinositol 3-kinase)/protein kinase B (AKT) cascade, which has contribution in the regulation of cell motility, survival, adhesion, and glucose metabolism, have been associated with the TC pathogenesis (11, 12). Chromosomal rearrangements (fusion genes) and point mutations are the two main molecular mechanisms associated with TC. A single nucleotide is altered in case of a point mutation, while 2 different genes are fused in case of chromosomal rearrangement. In most of the cases, these genetic alterations are somatic or nonhereditary in nature. Several endocrine neoplasia syndromes (such as- MEN2A, MEN2B) and familial forms of MTC involve hereditary germline mutations (12). Suspected thyroid tissues are analysed for potential somatic mutations. On the other hand, peripheral blood collected from the patients and perhaps their relatives are used to identify germline mutations (11, 13).

**Figure 3:**
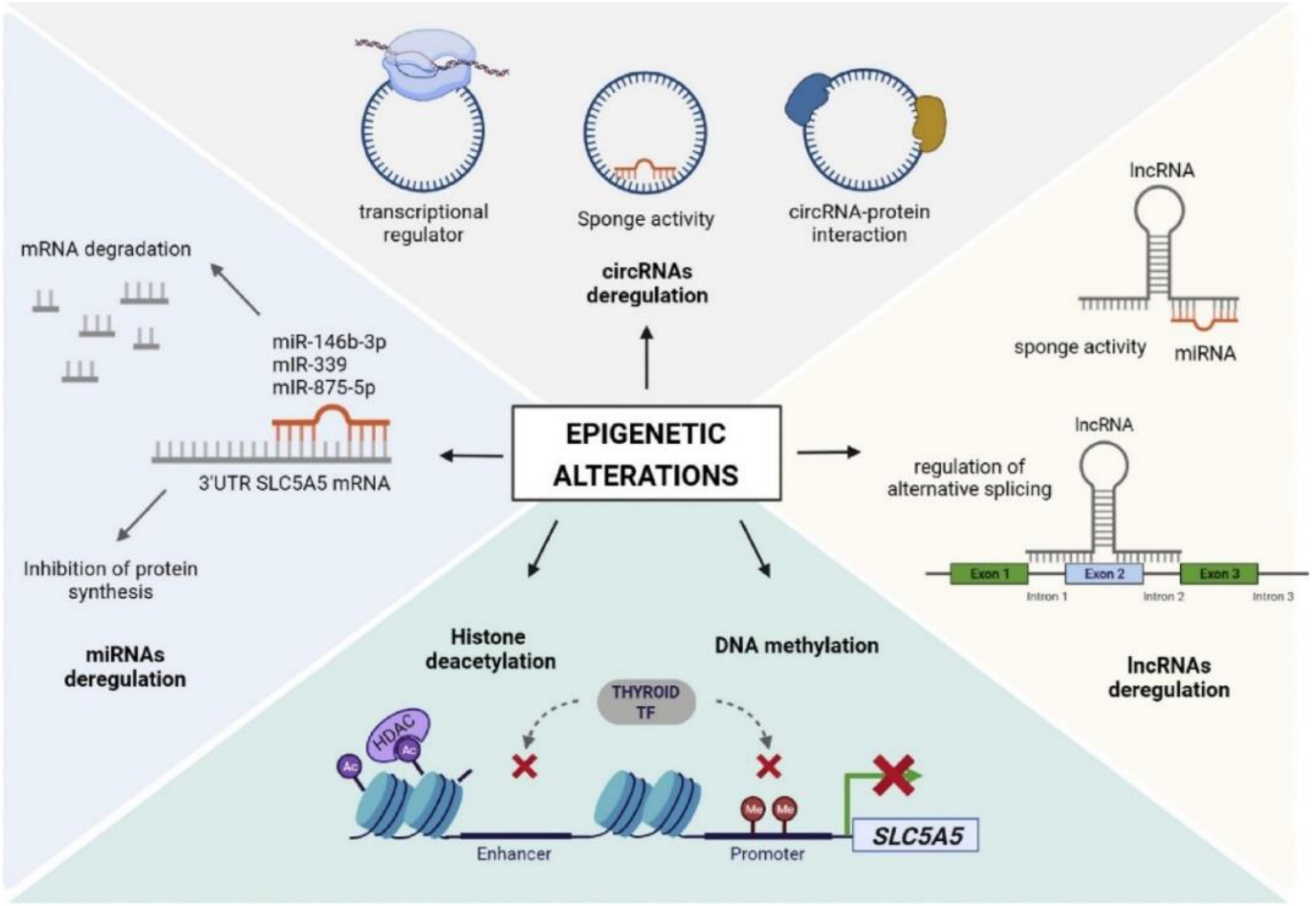
The role of epigenetic modifications in thyroid cancer. Reproduced with permission from Elsevier, (15).

Epigenetic processes are vital for the maintenance of tissue-specific gene expression patterns and normal development of cells in mammals (14). Nonetheless, epigenetic changes can lead to abnormal functions or suppression of several signalling cascades, which can eventually result in cancer including TC (**Figure 3**). Several studies have observed the link between epigenetic modification and a range of cancers, along with several genetic variations (16, 17). Epigenetic processes involve nucleosome remodelling, non-coding RNA expressions, DNA cytosine methylation, and covalent chromatin modification. Abnormal DNA methylation is linked with gene expression and has a contribution in tumorigenesis. Hypomethylation through several mechanisms can result in proto-oncogene activations and genomic instability, which can play a role in cancer progression and development. Nevertheless, hypermethylation is linked with gene silencing (predominantly tumour suppressor genes) and is regarded as a cancer hallmark. More studies regarding how certain genomic areas are targeted for hypermethylation are likely to lead to the development of more therapeutic areas. In addition, miRNA expression profile is another feature of epigenetic modification. Previously, miRNA expression profiles in tumours were compared to the related normal tissues, which indicated extensive expression level alterations. As microRNAs (miRNAs or miRs) control the expression levels of many genes that are associated with apoptosis, cell proliferation, and transcriptional regulation, therefore changes in their expression levels can mediate tumorigenesis. Indeed, miRNAs have the capacity to play a role as oncogenes or tumour suppressors, according to their role on the target genes.

## 3 Genetic modifications that can be considered as biomarkers in the diagnosis and prognosis of thyroid cancer

### 3.1 BRAF mutations

BRAF (7q24) is a proto-oncogene, which is responsible for encoding serine/threonine kinase belonging to the RAF-kinase family that has an important contribution in the signal transduction along the RAS/RAF/MEK/ERK cascade controlling apoptosis, differentiation, and cell growth. Among the 3 functional RAF proteins identified in humans including c-RAF, BRAF, and ARAF; BRAF exhibits the maximum basal kinase function and is the most strong MAPK cascade activator. Mutations in BRAF have also been linked with human carcinogenesis, where an increased level of BRAF mutations was observed in ovarian carcinoma, colorectal carcinoma, and melanomas. All such mutations were observed in the kinase domain of the protein, linking either the ATP binding site or activation loop, which further contributes in the activation of BRAF. Subsequently, they were also detected in PTCs and might be a target for potential therapy development against aggressive lesions (18). In the case of PTC, BRAF mutations are the most commonly detected mutations, along with the highest frequencies in classical PTC and tall-cell. BRAF mutations are exclusively observed in PTC and PTC-derived-ATC. So far, over 40 BRAF mutations have been detected, among them V600E is most commonly observed and responsible for around 95% of cases. BRAF K601E is most commonly seen in the case of follicular variant PTC (FV-PTC). Upon radiation exposure, a chromosomal rearrangement leading to the fusion gene AKAP9/BRAF and small in-frame deletions or insertions surrounding codon 600 were detected in PTCs. As compared to children, adults are more commonly affected by BRAF mutations. Fluorescence *in situ* hybridisation study revealed that BRAF activation includes copy number gain and is present in 35% of FTCs, 25% of follicular adenomas (FAs), and 3% of PTCs (18).

### 3.2 RAS mutations

Ras proteins belong to the guanosine triphosphate (GTP)-binding protein family that control cell growth via phosphoinositide-3-kinase (PIK3) and MAPK signalling pathways. In TC, 3 of RAS family including members NRAS (located at chromosome position 1p13), KRAS (located at chromosome position 12p12), and HRAS (located at chromosome position 11p11) were found to be mutated, wherein they mainly become activated either through mutations that reduce their intrinsic GTPase action (codon 61) or increase their GTP-binding affinity (codons12/13). Since their discovery, RAS mutations have been detected in 18–27% of poorly differentiated TCs (PDTCs), 10–20% of PTCs (particularly FV-PTC), up to 60% of ATCs, 40–50% of FTCs, and 20–40% of FAs. In general, the most common mutations are less common in codon 61 of HRAS and most common in codon 61 of NRAS. Furthermore, they are rarely observed in radiation-mediated TCs of Chernobyl and more commonly observed in iodine-deficient regions (18). RAS mutations were initially regarded as early events in thyroid carcinogenesis because of their occurrence in both FTCs and FAs, nonetheless this might be elucidated through the high extent of interobserver variability regarding the difference of FAs from FV-PTCs. It has been recently revealed that there is an increased level of RAS mutations in ATCs and PDTCs as well as lower levels of RAS mutations in well-differentiated TCs, which indicates the influence of RAS in the progression of the tumour, instead of the initiation. It has been demonstrated by *in vitro* studies that elevated levels of genomic instability mediated via RAS-genomic instability can mediate the progression of the tumour through permitting tumour cells to collect mutations that mediate elevated levels of invasiveness and survival. In addition, thyroid-targeted RAS mutation led to follicular tumours that further resulted in PDTCs in mice. RAS mutations might also be prognostic of poor prognosis in the case of PDTCs and well-differentiated TCs (18).

### 3.3 RET mutations

It is now well-known that the *RET* gene encodes a receptor tyrosine kinase and has a significant contribution in cell survival, differentiation, and growth (12). Typically, *RET* gene-associated point mutations are observed in MTC (19). Interestingly, 95% of MEN2B and MEN2A individuals contain germline mutations, while the occurrence of somatic mutations in sporadic MTC and germline mutations in familiar MTC is lower (50%) (20). For relatives of patients, genetic screening is suggested with an identified germline mutation in the RET gene (21). There is a high risk of MTC in case of an inherited *RET* mutation, where it is suggested to go through a preventive total thyroidectomy (22). Individual suggestions can be provided as per the identified genotype-phenotype correlations, particularly about the timing of prophylactic total thyroidectomy in childhood to avert the disease development (13, 23).

### 3.4 *RET/PTC* rearrangements

In children and adolescents, the most commonly observed mutation is the chromosomal rearrangement of *RET*/*PTC* (24). This kind of rearrangement is more common in PTC and is linked with frequent metastatic dissemination and more aggressive tumour behaviour (25). Carcinoma cases are observed following previous exposure to radiation (25). Identification of RET/PTC rearrangement serves as a strong indicator of PTC and might also facilitate the molecular diagnosis of FNAC, particularly in uncertain cytological findings. Total thyroidectomy is typically suggested following identification of *RET/PTC* rearrangements (13, 26).

### 3.5 Paired-box gene 8/Peroxisome proliferator-activated receptor-gamma fusion oncogene

A balanced translocation, t(2;3)(q13;p25), can lead to PAX8/PPARγ fusion oncogene that fuses PPARγ and PAX8. A translocation t(2;3;6;15) was first detected in an FA, which was then confirmed in FTCs and FAs. Subsequently, the presence of PPARγ and PAX8 was confirmed in this translocation and it was observed that fusion protein expression is induced by the PAX8 promoter. PAX8 plays an important role in regulating the terminal differentiation in thyroid cells, which regulates the expressions of thyroid-stimulating hormone (TSH) receptor, thyroglobulin, and sodium iodide symporter (NIS). Therefore, it is expected that the expression pattern of PAX8/PPARγ fusion protein is associated with the differentiation behaviour of thyroid tumours, where absent or low level is expected in poorly differentiated tumours and high level in well-differentiated tumours. On average, it has been reported that PAX8/PPARγ present in 13% of FV-PTC (0–50%), 11% of FAs (0–55%), and 36% of FTCs (0–63%). The aforementioned findings are based on the RT-PCR, however it should also consider the fact that promoters that drive expression of PAX8/PPARγ mRNA might be missing in the cells that are present in the dedifferentiated tumours, nonetheless still might harbour the fusion at the DNA level. In an attachment-independent manner, PAX8/PPARγ mediated *in vitro* thyroid cell growth, which decreased apoptosis and elevated soft agar colony formation (27). These effects might take place because of the fusion protein’s dominant negative suppressive action as compared to wild-type PPARγ, which is thought to possess tumour-suppressive activities and has been identified as a possible target for the development of therapeutics against various cancer types. The role of PPARγ in TC has been further revealed by the identification of another fusion protein in FTC, CREB3L2-PPARγ, which might arise a chromosomal rearrangement, t(3;7)(p25;q34). However, more studies are required to find out whether PAX8/PPARγ alone can mediate TC or whether additional epigenetic or genetic processes are needed to activate the full phenotypic expression of follicular TC (18).

### 3.6 *Telomerase Reverse Transcriptase* mutations

The rate-limiting catalytic subunit of telomerase is encoded by the *TERT* gene, which is accountable for the elongation of telomere during the replication of DNA (12). *C250T and C228T are the two main* point mutations that have been identified in *TERT* gene (28, 29). An increased level of telomerase expressions have been observed in cancer cells positive for *C250T and C228T* mutations, where these cancer cells can maintain chromosomal telomere length and can continue proliferation indefinitely (29). It has been observed that there is a link between *TERT* mutations and tumour aggressiveness as well as distant or local metastasis (28). *TERT* mutations are found in ATC, more aggressive forms of PTC, and poorly differentiated TCs (28, 30). Co-occurrence of *BRAF V600E* mutation and *TERT* mutations is linked with increased level of tumour aggressiveness in case of PTC as compared to *BRAF* and *TERT* mutations occurring alone (31). Region VI elective neck dissection and total thyroidectomy are recommended for the preoperative detection of *TERT* mutations in nodules bigger than 1 cm as per the European Thyroid Association (13, 26).

### 3.7 *Neurotrophic tyrosine receptor kinase* fusion genes

The occurrence of *NTRK* fusion genes in PTC is 5–10% in adolescent and pediatric patients (32). If a *NTRK* fusion gene is detected in a thyroid sample, the risk of malignancy is considered as 100% (21, 32). The follicular arrangement is observed in NTRK fusion-positive carcinomas, along with the incidence of frequent lymph node metastasis and chronic lymphocytic thyroiditis (13, 19).

### 3.8 *TP53* mutations

*TP53* gene (a tumour suppressor) starts apoptosis in the case of nonrepairable DNA and controls the cell growth by regulating the cell division (12). Increased levels of *TP53* expressions and mutations are identified in over 75% of undifferentiated and invasive carcinomas (21). In differentiated carcinomas, the occurrence of *TP53* mutations is considered as an indication of subsequent ATC dedifferentiation (19).

### 3.9 *Eukaryotic translation initiation factor 1A X-linked* mutations

The *EIF1AX* gene encodes a vital eukaryotic translation initiation factor and *EIF1AX* mutations have been linked to several cancers. Mutations in the *EIF1AX* gene have been detected in tumours that typically be deficient in other common drivers and identified in 1.5% of The Cancer Genome Atlas (TCGA) cohort, which are indicating that *EIF1AX* gene may play a role a novel PTC oncogene (33). *RAS* and *EIF1AX* mutations often co-occur, however the precise mechanism of action is yet to be discovered. These mutations are observed in benign neoplasms and in around 30% of PTC, which are closely linked with *TERT* and *RAS* mutations and indicate individuals in advanced TC with a reduced rate of disease-specific survival (34).

## 4 Epigenetic modifications that can be considered as biomarkers in the diagnosis and prognosis of thyroid cancer

### 4.1 Aberrant DNA methylation

Aberrant DNA methylation (ADM) of tumour suppressor genes and proto-oncogenes are found in TC and other human cancers. Tumour suppressor genes present in the thyroid include tissue inhibitor of metalloproteinase 3 (*TIMP3*), solute carrier family 5 member 8 (*SLC5A8*), Ras association domain family member 1, isoform A (*RASSF1A*), RAP1 GTPase activating protein (*RAP1GAP*), *RAPβ2*, phosphatase and tensin homolog (*PTEN*), and death associated protein kinase (DAPK). A class of GTPase-activating proteins is encoded by the *RAP1GAP* gene, which is responsible for the deactivation of RAS-related protein. This gene also controls mitogenic and oncogenic mechanisms in thyroid cells. On the other hand, RAP1 has a significant contribution in the ERK-dependent cascade regulation and BRAF-MEK-ERK cascade activation. In thyroid tumour cell lines, the immunohistochemistry studies showed *RAP1GAP* gene downregulation in PTC along with its invasion and proliferation. *PTEN* is a tumour-suppressive gene, which is responsible for encoding phosphatidylinositol-3, 4, 5-triphosphate 3-phosphatase protein. *PTEN* mutations have been detected in several cancer types. *PTEN* gene is also responsible for the negative regulation of AKT/protein kinase B (PKB) signalling cascade. Moreover, this gene has a significant contribution in controlling cell cycle and opposing rapid cell division and growth. ADM of *PTEN* is commonly observed in both PTC and FTC.

The *TIMP3* gene is responsible for the suppression of cell development, angiogenesis, infiltration, and metastasis of many tumours. A hypermethylation of *TIMP3* gene has been detected in the case of TC. This gene is also linked with extrathyroidal invasion and lymph node metastasis (35). A protein analogue to the RAS effectors protein is encoded by the *RASSF1A* gene. A link has been reported between the cancer and *RASSF1A* mRNA expression deregulations, where ADM plays an important in the inactivation of *RASSF1A* gene. Unlike FTC, ADM of *RASSF1A* is present in a small proportion in PTC, which might have a significant contribution in thyroid carcinogenesis. DNA hypomethylation also has a significant contribution in carcinogenesis, however its exact role is yet to be fully revealed. Nonetheless, international patterns of ADM have been revealed in subtypes of thyroid malignancy through DNA methylation arrays. Collectively, 13 and 21 hypomethylated genes in FTC as well as 262 and 352 hypermethylated genes in PTC have been identified. Moreover, 86 and 131 hypermethylated as well as 280 and 393 hypomethylated genes were detected in MTC and ATC, respectively. Out of these genes, 4 oncogenes including TCL1B, NOTCH4, INSL4, and DPPA2 were found to be commonly controlled by hypomethylation (35).

### 4.2 Histone modifications

The link between the behaviour of thyroid tumours and histone modifications has been demonstrated. It is well-established that the gene transcription is dependent on the chromatin accessibility and conformation. Chromatin remains “open” (euchromatin) in a transcriptionally active state, which permits the transcriptional machinery interaction with DNA to initiate the transcription of genes, while DNA remains tightly wrapped around the closed state of chromatin (heterochromatin). On the other hand, DNA tight packaging is caused by its coiling around histone proteins, which provide structural support to a chromatin. Through de-acetylation and acetylation of lysine residues, the extent of DNA condensation around histones regulates gene transcription, which involves histone post-translational modification. Histone deacetylases and acetyltransferases are the enzymes that cause such reversible acetylation-deacetylation alterations. The interaction of DNA and histones is hindered by histone acetylation of lysine residues through the removal of the positive charge on the histones that is responsible for the interaction with the negative charge containing phosphate groups of DNA. The role of histone acetylation in cancer has been widely evaluated, where this acetylation has a significant contribution in tumorigenesis.

Histone acetylation in TC plays a role from the early stages of thyroid carcinogenesis. Increased concentrations of H3K9–K14ac and H3K18ac have been reported in FTC and PTC than in control tissues, while histone H3K9–K14ac was only identified in ATC tissues, further indicates that the deficiency of the expression of H3K18Ac in case of ATC might have contribution during the progression of TC (36). It was observed that ectopic induction of major driver oncogenes including RAS, BRAF or RET/PTC resulted in elevated concentrations of acetylated histones in thyroid cell lines, which is in contrast with the events that take place in undifferentiated and advanced tumours where acetylation decreases expressions of a range of thyroid differentiation genes including *NKX2.1* (NK2 Homeobox 1), *TPO* (thyroid peroxidase), TG (*Thyroglobulin*), and *SLC5A5* (solute carrier family 5 member 5) (37). HRAS and BRAF^V600E^ mutations (unlike other genetic modifications) exhibit less response to histone deacetylase (HDAC) inhibitor therapies regardless of thyroid tumour subtypes. An improved antitumour action has been observed in some tumour cell lines with the combined treatment of PI3K/Akt or MAPK inhibitors and HDAC inhibitors. Panobinostat is a strong inhibitor of HDAC that exerted *in vitro* and *in vivo* cytotoxic actions on ATC cell lines, which resulted in apoptosis as well as cell cycle arrest and blocked growth of tumours in a xenograft mouse model. Treatment with panobinostat also re-stimulated mRNA expression of SLC5A5 along with an increased level of NIS, these effects were also demonstrated with two other inhibitors of HDAC trichostatin and suberoylanilide hydroxamic acid (38). Several clinical trials were carried out utilising various HDAC inhibitors including depsipeptide, valproic acid, romidepsin, vorinostat, and suberoylanilide hydroxamic acid (39, 40). Nonetheless, these clinical trials failed to show promising outcomes, in spite of the great potential of these HDAC inhibitors (15).

### 4.3 Non-coding RNAs

#### 4.3.1 Long non-coding RNAs

Typically, lncRNAs are described as transcripts that contain over 200 nucleotides that are not generally translated into functional proteins. In addition, they are commonly found in the nucleus, wherein lncRNAs exhibit various activities including gene expression and splicing regulation via various mechanisms. Chromatin structure can be altered due to the interaction of lncRNAs with DNA, which can result in epigenetic modifications and further cause alterations in the target gene expressions. In addition, lncRNAs can interact with miRNAs or mRNAs and play a role as molecular sponges or competing endogenous RNAs (ceRNAs) to control the miRNA interaction with the targets or to regulate the translation and stability of mRNAs. A range of deregulated lncRNAs have already been reported, which can play role as biomarkers in the prognosis and diagnosis of TC (**Table 1**). For instance, HOX transcript antisense RNA (HOTAIR) is a lncRNA that is commonly overexpressed, which was found to be overexpressed in patients with PTC and TC (41). HOTAIR is commonly linked with survival and it contributes in thyroid carcinogenesis through Wnt signalling (42). In a miR-1-induced manner, silencing of HOTAIR markedly decreased the tumour growth *in vivo* as well as the growth of FTC-133 and TPC-1 cell lines via controlling the expression of CCND2. Overexpression of nuclear-enriched Abundant Transcript 1 (NEAT1) has been reported in case of PTC and NEAT1 silencing in TPC-1 cells resulted in decreased *in vivo* tumour growth, motility, and cell survival induced by the downregulation of β-catenin through the regulation of miR-214 (54). Trinucleotide Repeat Containing Adaptor 6C-Anti Sense1 (TNRC6C-AS1) is another lncRNA that plays a role as an oncogene in TC via sponging miR-129-5p as well as mediating invasion, proliferation, and migration of TPC-1 cells (55). TNRC6C overexpression or TNRC6C-AS1 silencing re-induced the expressions of various thyroid genes including *TPO*, *TSHR* (thyroid stimulating hormone receptor), *SLC26A4*, and *SLC5A5* (56). Therefore, it is likely that the TNRC6C-AS1–TNRC6C axis has a contribution in iodine metabolism regulation in case of PTC. Overexpression of *MALAT1* (metastasis-associated lung adenocarcinoma transcript 1) has also been observed in PTC, where it exerts oncogenic activities (57). Nonetheless, downregulation of MALAT1 has been reported in ATC and PDTC (57). In the case of TC, *MALAT1* mediates invasion and proliferation of cells via IQGAP1 upregulation (58), which is a vital MAPK scaffold protein that has significant contribution in TC (59).

**Table 1:** Summary of potential lncRNAs as diagnostic and prognostic biomarkers in thyroid cancer.

lncRNAs	Type of thyroid cancer	Target	Roles	References
HOX transcript antisense RNA (HOTAIR)	Papillary thyroid cancer (PTC)	miR-488-5p	Regulates the disease progression and tumorigenesis of PTC via controlling the cellular malignancy	(41, 42)
Nuclear-enriched Abundant Transcript 1 (NEAT1)	PTC	miR-524-5p	NEAT1 elevates histone deacetylase 1 gene (HDAC1) expression via sponging miR-524-5p	(43)
TNRC6C-AS1	Thyroid cancer (TC)	miR-513c-5p	TNRC6C-AS1 suppresses apoptosis and autophagy of TC cells via STK4 methylation by using Hippo signalling pathway	(44)
Metastasis Associated Lung Adenocarcinoma Transcript 1 (MALAT1)	Anaplastic TC (ATC)	miR-200-3p	Associated with the autophagy, invasion, migration, apoptosis, and cell proliferation	(45)
Long Intergenic Non-Protein Coding RNA 313 (LINC00313)	PTC	miR-4429	Regulates the migration and proliferation of PTC cells	(46)
AB074169	PTC	KH-Type Splicing Regulatory Protein (KHSRP)	AB074169 controls cell proliferation through modulating KHSRP-induced CDKN1a expression	(47)
Taurine upregulated gene 1 (TUG1)	TC	miR-145	Elevated TUG1 expression significantly induces tumor cell invasion and proliferation	(48)
ZNFX1 Antisense RNA 1 (ZFAS1)	PTC	miR-590-3p	ZFAS1 overexpression can mediate proliferation and suppress apoptosis of PTC cells	(49)
Actin filamentin-1 antisense RNA 1 (AFAP1-AS1)	ATC	miR-155-5p	Overexpression of AFAP1-AS1 leads to migration, proliferation, invasion and apoptosis inhibition of tumor cells	(50)
Long Intergenic Non-Protein Coding RNA 313 (LINC00313)	TC	ALX Homeobox 4 (ALX4)	Regulates cell invasion, migration, and proliferation	(4)
BRAF-Activated Non-protein Coding RN (BANCR)	PTC	Thyroid stimulating hormone receptor (TSHR)	BANCR mediates cell proliferation in PTC	(51)
HOXA Cluster Antisense RNA 2 (HOXA-AS2)	PTC	miR-520c-3p	HOXA-A52 mediates cell invasion and migration	(52)
LOC100129940-N	PTC	Wnt/*β*-catenin signalling	Mediates cell invasion, migration, and proliferation	(53)

The expression of *MALAT1* is controlled via TGFβ, which indicates its contribution in TC progression by processes associated with epithelial-mesenchymal transition (60). Interestingly, some lncRNAs act as tumour suppressors via controlling epithelial differentiation and cell homeostasis. For example, in PTC, *CASC2* (Cancer Susceptibility Candidate 2) was found to be downregulated in samples and its lower level was linked with poor prognosis (60). CASC2 overexpression markedly decreased *in vitro* cell proliferation and resulted in ERK1/2 and AKT inactivation (60, 61). Furthermore, CASC2 suppressed the invasion and migration of TC cells via sponging miR-18a-5p and miR-155 (62). GAS5 (Growth Arrest-Specific 5) is another lncRNA and its downregulation was observed in TC cell lines and PTC in contrast with samples containing benign tumour. In patients with PTC, a lower level of GAS5 expression is linked with poor prognosis, tumour nodules metastasis (TNM) staging, multiple cancer foci, and lymph node metastasis (63). In PTC cell lines, GAS5 played a role as a ceRNA of miR-222-3p and inactivated the PI3K signalling caused by the upregulation of PTEN (63). These findings indicate the role as a tumour suppressor in TC. *LINC00893* (Long Intergenic Non-Protein Cocing RNA 893) is another lncRNA that is linked with PI3K signalling through PTEN signalling. Reduced expression of *LINC00893* has been reported in TC and PTC cells (64). In PTC cell lines, *LINC00893* ectopic overexpression diminished cell migration and proliferation via AKT phosphorylation blockage (64).

#### 4.3.2 Circular RNAs

Numerous studies have already discovered the link between circRNAs and tumorigenesis. The circRNAs play a role as a ceRNA to control gene expression via suppressing miRNA activities (**Table 2**). There is a growing interest regarding the functions and roles of circRNAs. In a study, 98 deregulated circRNAs were detected when comparing six PTC tumours with nearby normal tissues (70). Among them, circRNA-102171 overexpression was observed in 47 PTC samples as compared to contralateral healthy tissues (71). In a different study, 146 deregulated circRNAs were detected after RNA sequencing of 11 PTCs and their heterolateral non-tumour tissues (72). On the other hand, circ_0011058 is another circRNA that mediated *in vivo* tumour growth and angiogenesis and facilitated cell proliferation in PTC cell lines via controlling YAP1 expression and sponging miR-355-5p (73). An upregulated level of CircRUNX1 was observed in cancer samples to control the miR-296-3p/DDHD2 axis associated with metastasis formation and tumour growth (74). Additional activities of circRNAs include altering protein and gene expression by controlling gene transcription, through their role as a translation template or by interacting with the transcription machinery. Indeed, circRNAs also have the capacity to modify the localisation or functions of the proteins via interactions. For instance, circ-Amotl1 can interact with c-MYC to activate and stabilise its transcriptional function through the regulation of its nuclear translocation (75). Furthermore, circRNA_102171 activates the Wnt/β-catenin signalling through direct interaction with CTNNBIP1, which eventually mediates the invasiveness of PTC cells (71).

**Table 2:** Summary of potential circRNAs as diagnostic and prognostic biomarkers in thyroid cancer.

circRNAs	Type of thyroid cancer	Target	Roles	References
Circ_100395	Papillary thyroid cancer (PTC)	Phosphatidylinositol 3-kinase (PI3K)/protein kinase B (AKT)/mammalian target of rapamycin (mTOR) signalling	Circ_100395 overexpression markedly decreased cell invasion, migration and survival via the PI3K/AKT/mTOR signalling pathway	(65)
hsa-circ-u0058124	PTC	miR-218–5p	Mediates cell metastasis, tumour invasiveness, tumourigenicity, and proliferation	(66)
CircNEK6	PTC	miR-370-3p	Promotes PTC progression	(67)
circ-ITCH	PTC	miR-22-3p	circ-ITCH is associated with sponging miR-22-3p and elevation of CBL expression	(68)
circ-BACH2	PTC	miR-139-5p	Mediates PTC cell invasion, migration, and growth	(69)

#### 4.3.3 MicroRNAs

Among the ncRNAs, miRNAs have been best characterised and most studied. These ncRNAs contain RNA transcripts of 18–24 nucleotides that interact with the 3′-UTR of mRNAs to hinder protein translation of target genes. They have a significant role in cancers, where miRNAs play a role in enhancing tumour progression and loss of differentiation. They can be classified as tumour suppressors and oncogenic (oncomiRs) as per their activities on death and proliferation of cells as well as expression patterns in malignant samples in contrast with healthy tissues. Certain profiles of miRNA expressions can be linked with genetic mutations commonly detected in DTCs. Moreover, miRNAs can be easily detected in blood samples and they show resistance to various environmental conditions including room temperature, therefore a miRNA profile can be used as therapeutic targets and prognostic biomarkers (**Table 3**). In contrast with circulating mRNAs, miRNAs have the ability to remain protected from nucleases in the bloodstream by being encased in exosomes or microvesicles or through interaction with proteins as well as miRNAs have the capacity to remain undamaged in paraffin-fixed tissue samples (85, 86). Numerous studies have already assessed the role of various miRNAs in thyroid carcinogenesis, where they regulate the major cancer-associated signalling mechanisms including transforming growth factor beta (TGFβ), PI3K, MAPK, Hippo and Wnt signalling mechanisms (15, 87). PTC miRNAs are classified into 6 clusters as per TCGA that are markedly different as per the parameters including risk profiles, histological phenotype, driver mutations, and so on. For example, Cluster 1 is closely linked with RAS mutations and FV-PTC, while clusters- 5 and 6 contain the highest level of BRAFV600E mutations and a very high risk.

**Table 3:** Summary of potential miRNAs as diagnostic and prognostic biomarkers in thyroid cancer.

miRNAs	Type of thyroid cancer	Target	Roles	References
miR-221	Papillary thyroid cancer (PTC)	CDKN1B/p27	Affects the cell cycle and the p27 protein level	(76)
miR-222	PTC	CDKN1B/p27	Regulates cell cycle and p27 protein level	(77)
miR-137	PTC	CXCL12	miR-137 suppresses PTC cell invasion, migration, and proliferation	(78)
miR-146b	PTC	Epidermal growth factor receptor (EGFR), nuclear factor-κB (NF-κB), interleukin 1 receptor-associated kinase 1 (IRAK1), and SMAD family member 4 (SMAD4)	Expression of miR-146b was positively linked with cell invasion, migration, and proliferation	(79)
miR-206	PTC	MAP4K3	miR-206 upregulation suppressed the cell proliferation and stimulated apoptosis	(80)
miR-486	PTC	Fibrillin-1 and KIAA1199 (Cell migration inducing protein or CEMIP)	Regulates PTC cell invasion and metastasis	(81)
miR-1179	PTC	High Mobility Group Box 1 (HMGB1)	Regulates PTC progression	(82)
miR-1266	PTC	Fibroblast growth factor receptor 2 (FGFR2)	miR-1266 overexpression in PTC cells markedly decreased cell invasion, migration, and proliferation	(83)
miR-25-3-p	PTC and follicular thyroid cancer (FTC)	Suppressor Of Cytokine Signalling 4 (SOCS4)	Increases cell invasion and metastasis	(84)

On the other hand, Cluster 6 possesses most of the tall-cell PTC samples (74%) of TCGA (88). As like FTC tumours, FV-PTC exhibits a RAS-like signature, however some of the most deregulated miRNAs are common in PTC (76, 89), unlike TCGA findings where the expression patterns of miRNA of cluster 1 were markedly dissimilar to the most deregulated miRNA in PTC (15).

Unlike PTC, FTC shows a specific and different pattern of miRNA expression, however there are a minimum 2 shared miRNAs including mir-221 and miR-34 that have significant contributions in well-differentiated tumours in case of thyroid carcinogenesis (76). On the other hand, ATC contains several deregulated miRNAs present in FTC and PTC, however it contains specific deregulated miRNAs that are associated with the malignancy and dedifferentiation (90, 91). ATC also contains heterogenous cell types and a higher level of downregulated miRNAs (91). Collectively, these findings confirm the significance of epigenetics as a diagnostic tool in the case of TC. Numerous studies have already analysed and identified miRNA profiles differentiating benign from malignant thyroid neoplasms, which could be used by clinicians in postoperative monitoring. A combination of quantitative reverse transcription polymerase chain reaction (RT-PCR) and sequencing showed overexpressed levels of miR-222 and miR-151-5p in tissue and serum samples of PTC patients in comparison with healthy controls and goitre patients (77). Levels of miR-222 and miR-151-5p were found to be reduced following thyroidectomy compared to the levels observed in healthy people (92–94). A range of other PTC-linked miRNAs including miRNA-146b, miR-451a, miR-25-3p, miRNA-190, miR-29b, miRNA-95, and miRNA-579 have been identified.

Indeed, miR-146b is a well-studied and most overexpressed miRNAs in TC, which is most commonly seen in PTC. The miR-146b expression is closely linked with the malignant thyroid neoplasm occurrence, which makes this miRNA an important biomarker (79). It was observed that miR-146b-3p can interact with the 3′-UTR of SLC5A5 and PAX8, which further leads to iodide uptake and protein translation inhibition (95). It has been observed that miR-146b-5p interacts with 3′-UTR of PTEN and SMAD4 (SMAD Family Member 4) to decrease the level of mRNA expression and stimulate PI3K/AKT signalling mechanism and TGFβ hyperactivation, which eventually leads to an elevated level of cell aggressiveness (96, 97). In addition, miR-146b-5p decreases the biosynthesis of miRNA via targeting DICER1, which is an important protein required for miRNA maturation that contributes as a tumour suppressor in TC (98). Both cell proliferation as well as invasion are regulated by miR-146b-5p and these processes are up-regulated throughout the epithelial-mesenchymal transition, therefore affects the progression of PTC. Several studies already reported the potential of miRNA profiles (including miR-146b) for the accurate differentiation benign as well as malignant tumours in fine-needle aspiration biopsy (FNAB) specimens (99–102). Levels of circulating miR-146b have been designated as an important and reliable serological marker for differentiating between benign tumours and PTC (103). Identification of the expressions of miR-146b in case of several thyroid nodules via using *in situ* hybridisation study confirmed its significance in differentiating between PTC from anaplastic TC, FTC, follicular adenomas, or poorly differentiated TC (84, 104). Increased concentrations of miR-21 and miR-146b-5p were also linked with markedly lower survival rates in PTC patients. In PTC, mir-146b-5p has been suggested as a prognostic and diagnostic marker because of its increased expression level in PTC unlike other tissues examined (104).

TCGA carried out a study with TC samples, which observed that miR-146b-5p-induced regulation of the *IRAK1* (interleukin-1 receptor-associated kinase 1) gene which is different from the conventional form of PTC (105). NF-κB/IL6/STAT3 signalling cascade was also found to be linked with the regulation of synthesis of miR-146b-5p, whose elevated concentrations downregulate the expressions of various pro-inflammatory mediators including IRAK1 (106). The deregulation of miR-146b was linked with aggressive behaviour of tumours in clinical PTC specimens positive for *BRAF* and individuals containing *BRAF* mutations showed elevated expressions of miR-146b as compared to *BRAF* wild-type controls (107, 108). Various other miRNAs including miR-17-92, miR-339, and miR-875-5p cluster also have the capacity to interact with the 3′UTR of SLC5A5, which further decreases the expressions of NIS. An increased concentration of miR-875-5p expression was detected in the case of PDTC, while miR-339 expression was less commonly observed (109). Elevated levels of miR-17-92 expression decreased PAX8 and other genes responsible for iodine metabolism (110), whereas the miR-29a level is downregulated and targets LOX gene (91). An elevated level of PI3K cascade regulation by microRNAs has been observed in TC. PTEN is also targeted by miR-21, where an increased expression has been observed in various solid tumours including PTC. The expression of miR-21 is controlled through RAS by the activator Protein-1 transcription factor and minimum 2 downstream pathways including PI3K and MAPK (111, 112). Both miR-222 and miR-221 can interact with the 2 target regions in the 3′-UTR area of the p27-kip1 transcript, an established downstream effector of the PI3K cascade and a negative cell cycle regulator, which decreases its protein concentrations (112). The overexpression of miR-221 triggered cell invasion, migration and proliferation in various PTC cell lines (113).

An overexpression of miR-29 and miR-23 has also been reported, which are found to be controlled by TSH and its target SMAD3 (a key regulator of TGFβ activities) (114). Among the TC-related downregulated miRNAs, the let-7 miRNA family has a significant contribution in the development and control of cell fate. Members of the let-7 miRNA family have the capacity to interact with the 3′-UTR of all 3 RAS genes (including KRAS, NRAS and HRAS) via several binding regions, which contribute in decreasing the levels of protein expression. An activation of the RAS-ERK cascade was detected following the downregulation of let-7 miRNAs. A downregulation of let-7f was observed in PTC (115), while its expression was found to be linked with the levels of RAS protein (116). Moreover, a stable let-7f transfection in TPC-1 (the human PTC cell line) containing RET/PTC rearrangement resulted in the decreased cell proliferation and MAPK activation.

As per TCGA findings, expression of miR-137 is not markedly deregulated in TC, however a lower level of expression was observed in PTC as compared to adjacent normal tissues (117). It was observed that miR-137 can directly target the transcript of the tyrosine kinase receptor gene *EGFR* (epidermal growth factor receptor), which has a significant contribution in the MAPK signalling translocation from the membrane to the nucleus in order to cause activation of the major effectors of MAPK. In addition, miR-137 downregulated cell invasion, colony formation ability, and cell proliferation as well as negatively regulated ERK and Akt signalling pathways in TPC-1 and B-CPAP (the PTC cell lines). The reduction of EGFR repealed the action of miR-137 suppression on these signalling cascades, which indicates that miR-137 contributes on signalling in an EGFR-dependent manner. In a study, Nieto et al. used combined miRNA and mRNA expression as prognostic indicators of TC recurrence (78). They formulated a risk score model as per the comprehensive bioinformatics and experimental miRNA, mRNA, and somatic mutation study in recurrent tumours. Furthermore, they utilised RNA sequencing results of 501 TC samples obtained from TCGA datasets, including 46 recurrent tumours and 455 non-recurrent samples. These researchers also carried out a functional gene analysis in several thyroid cell lines in cell-based assays and evaluated the prognostic values of the genes by utilising the TCGA datasets (78). In total, they detected 59 genetic variants, 39 miRNAs, and 40 mRNAs as important biomarkers of TC recurrence. Among them, miR-1179 and miR-486 showed marked activities in suppressing *in vitro* TC cell migration, while deletion of miR-1179 and miR-486 elevated cellular migration *in vitro* (82, 118). A markedly higher level of miR-375 overexpression was observed in the case of MTC in comparison with the normal thyroid tissues. Moreover, there is a close connection between patient outcomes, expression of miR-375, and tumour aggressiveness, which indicates an important role in the pathogenesis of MTC (119, 120). Therefore, levels of circulating miR-375 are regarded as important prognostic markers for advanced MTC (121). In addition to this, serum miR-375 can be used as prognostic and diagnostic markers of MTC, which differentiates between control subjects and MTC patients along with a 97.6% specificity and a 92.6% sensitivity (122).

## 5 Conclusion and future perspectives

Advances in genetic research and the epigenetics revolution have extensively evaluated Over the past decades to find out whether genetic codes predominately determine gene function or not. Various experiments have also established the involvement of genetic and epigenetic modifications in cancer, which indicates that genome packaging is also vital as like genome in controlling the major cellular mechanisms. Therefore, a comprehension regarding genetic and epigenetic modifications is not only essential for the diagnosis and prognosis of various cancers including TC but also for the development of therapeutics. As like any other cancers, most of the genetic and epigenetic modifications in the case of TC are somatic in nature, thus evaluation of the epigenetic pattern in TC exhibited an important contribution in these modifications in the prognosis and classification of tumours. Interestingly, TC-associated epigenetic alterations are reversible; therefore it is possible to develop an optional epigenetic therapy. As miRNAs have substantial contribution in cell invasion, differentiation and proliferation, thus miRNAs as well as target genes can be used as potential targets for the diagnosis and treatment of tumours. Indeed, whole genome sequencing methods can extraordinarily identify the genetic lesions accountable for the dedifferentiation, progression and onset of TC. The molecular pathogenesis of TC has been changed owing to the growing knowledge of genomics and epigenomics. This improved understanding of TC-associated signalling mechanisms and complex intracellular networks has resulted in clinical trials with small kinase inhibitors.

Furthermore, mutation identifications in novel genes led to the detection of potential and novel molecular markers of TC. An updated classification of thyroid tumours as per the RAS-and BRAF-score or differentiation-score has mediated the development of precise molecular classification of these tumours. Novel findings on histone acetylation and DNA methylation might result in the detection of repressive molecules of both modifications that may further facilitate the thyroid tumour re-differentiation, which can further decrease their aggressive behaviours and their refractoriness to radioactive iodine. Collectively, all these findings have improved the knowledge regarding TC pathogenesis and its causes. Moreover, this knowledge has provided insights regarding the biological mechanisms linked with the progression and initiation of TC, regulatory circuits, new targetable cancer genes, and molecular markers with clinical uses in prognosis as well as diagnosis of TC.
